# Overall Survival Prediction in Glioblastoma With Radiomic Features Using Machine Learning

**DOI:** 10.3389/fncom.2020.00061

**Published:** 2020-08-04

**Authors:** Ujjwal Baid, Swapnil U. Rane, Sanjay Talbar, Sudeep Gupta, Meenakshi H. Thakur, Aliasgar Moiyadi, Abhishek Mahajan

**Affiliations:** ^1^Department of Electronics and Telecommunication Engineering, Shri Guru Gobind Singhji Institute of Engineering and Technology, Nanded, India; ^2^Department of Pathology, Tata Memorial Centre, ACTREC, HBNI, Navi-Mumbai, India; ^3^Department of Medical Oncology, Tata Memorial Centre, ACTREC, HBNI, Navi-Mumbai, India; ^4^Department of Radiodiagnosis and Imaging, Tata Memorial Centre, Tata Memorial Hospital, HBNI, Mumbai, India; ^5^Department of Neurosurgery Services, Tata Memorial Centre, Tata Memorial Hospital, HBNI, Mumbai, India

**Keywords:** brain tumor, glioblastoma, overall survival, radiomic, machine learning

## Abstract

Glioblastoma is a WHO grade IV brain tumor, which leads to poor overall survival (OS) of patients. For precise surgical and treatment planning, OS prediction of glioblastoma (GBM) patients is highly desired by clinicians and oncologists. Radiomic research attempts at predicting disease prognosis, thus providing beneficial information for personalized treatment from a variety of imaging features extracted from multiple MR images. In this study, first-order, intensity-based volume and shape-based and textural radiomic features are extracted from fluid-attenuated inversion recovery (FLAIR) and T1ce MRI data. The region of interest is further decomposed with stationary wavelet transform with low-pass and high-pass filtering. Further, radiomic features are extracted on these decomposed images, which helped in acquiring the directional information. The efficiency of the proposed algorithm is evaluated on Brain Tumor Segmentation (BraTS) challenge training, validation, and test datasets. The proposed approach achieved 0.695, 0.571, and 0.558 on BraTS training, validation, and test datasets. The proposed approach secured the third position in BraTS 2018 challenge for the OS prediction task.

## Introduction

Glioblastoma (GBM) remains the most aggressive primary malignant brain tumor in adults, with a median survival time of 15 months and 5-year survival of ~5% after initial diagnosis (Chang et al., 2016). Nearly all patients with GBM relapse despite providing maximal safe surgical resection, radiotherapy, temozolomide, and aggressive therapy. Spatial and temporal intra-tumor heterogeneity, extent, and location are some of the factors that make these tumors challenging to resect and, in some cases, inoperable. The inability to perform complete surgical tumor resection and poor drug delivery to the brain contributes notably to the lack of effective treatment and poor prognosis (Mahajan et al., 2015).

Certain biological variables such as MGMT promoter methylation status, 1p/19q deletion, and IDH1 gene mutation status have been shown to explain to a certain extent this observed variation, in addition to certain host variables such as age and gender. The fact that GBM shows extremely wide clinical behavior points to the fact that the current understanding of GBM as a single disease entity is an oversimplification. This is further supported by the fact that there have been multiple attempts to divide GBM into more distinct subgroups using molecular subtyping (Verhaak et al., 2010). However, these methods are difficult to replicate in routine clinical practice owing to the complexity of the assays and high costs. Further, tumors show subtype plasticity with a complex transition from one subtype to another during progression (Lee et al., 2018). Thus, predicting survival of patients with GBM is a challenging task.

Magnetic resonance imaging (MRI) plays a vital role in neuro-oncology for initial diagnosis and assessment of treatment response and is increasingly used as a powerful non-invasive predictive tool. Researchers have identified that MRI provides distinct information that can predict survival independently of pathologic and clinical data. The process that extracts various quantitative features on the basis of intensity, volume, shape, and textural variations from radiographic images and design predictive algorithms to find the association of these vast features to the survival and outcome of the patient is known as radiomics (Chaddad et al., 2019b). Radiomics incorporates several essential disciplines, including radiology for imaging interpretation, computer vision for quantitative feature extraction, and machine learning for classifier evaluation and regression (Seow et al., 2018; Vaidya et al., 2019).

In recent years, several radiomic models have been proposed for survival prediction (Huang et al., 2016), distant metastasis prediction (Coroller et al., 2015), and molecular characteristics classification (Kickingereder et al., 2016a,b). Researchers extracted several radiomic features on the basis of texture, area, volume, and Euler characteristics-based features from different intra-tumor parts (Shboul et al., 2019). Extreme Gradient Boosting (XGBoost) was used as a regressor to predict the OS. This approach achieved 0.519 accuracy on Brain Tumor Segmentation (BraTS) 2018 test dataset. In another study, multi-planer spatial convolutional neural networks were used for brain tumor segmentation, and semantic and agnostic features were extracted on these segmented tumor parts. These radiomic features were provided as input to multilayer perceptron (MLP) to predict OS (Banerjee et al., 2019). Although the proposed approach performed well for segmentation task, the algorithm performed poorly on BraTS 2018 test dataset for overall survival (OS) prediction task. Other than the sophisticated machine learning approaches, a simple linear regressor was used on only nine features. These features were computed by the volume, by summing up the voxels and the surface area, and by summing up the magnitude of the gradients along with three directions. There were fewer chances of overfitting because of only nine features, and hence, the method performed well (Feng et al., 2019). Multi-scale texture features-based approach for predicting GBM patients' progression-free survival and OS on T1- and T2-weighted fluid-attenuated inversion recovery (FLAIR) MRIs was proposed using the random forest (Chaddad et al., 2018). The study results showed that the identified seven-feature set, when combined with clinical factors, improved the model performance, yielding an area under the receiver operating characteristic curve (AUC) value of 85.54% for OS predictions. Osman et al. extracted a set of 147 radiomic image features locally from three tumor subregions on standardized preoperative multiparametric MR images. LASSO regression was applied for identifying an informative subset of chosen features, whereas a Cox model was used to obtain the coefficients of those selected features (Osman, 2019). Despite the various correlations between imaging features, genomic expression, and survival reported in the literature, no single analysis has been substantive enough to enter clinical practice.

In another study, usefulness of geometric shape features, extracted from MR images, as a potential non-invasive way to characterize GBM tumors and predict the OS times of patients with GBM, is evaluated (Chaddad et al., 2016a). Multi-contrast MRI texture features were used for the prediction of survival of patients GBM using texture features derived from gray-level co-occurrence matrices (GLCMs). The statistical analysis based on the Kaplan–Meier method and log-rank test was conducted in order to identify the texture features most closely associated with the OS (Chaddad and Tanougast, 2016; Chaddad et al., 2016b). A study underlines that radiomic features could be complimentary to biopsy-based sequencing methods to predict survival of patients with IDH1 wild-type GBM (Chaddad et al., 2019a).

This study aims to evaluate the efficiency of the radiomic feature-based MRI signatures from multi-modal MRI data and to find their associations with OS in patients with high-grade gliomas (HGGs) with improved accuracy compared with those of the available state-of-the-art methods. The rest of the manuscript is organized as follows: the dataset used for the study, preprocessing steps, and radiomic feature extraction framework is described in the *Material and Method*. Sample results are discussed in the *Result and Discussion*. *Conclusion and Future Work* concludes the paper with future direction.

## Materials and Methods

We participated in BraTS 2018 challenge, which mainly focused on two tasks:

1. segmentation of brain tumor with intra-tumor parts like edema, enhancing tumor, and necrotic part; and
2. OS prediction of the patients in days with the help of imaging features.

In this study, we mainly focused on the survival prediction aspects of the BraTS challenge.

### Dataset

**Figure 1:**
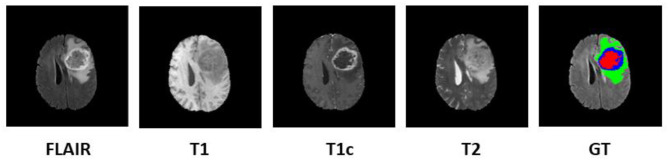
Multi-modal data with four channels provided in BraTS 2018 challenge dataset along with ground truth (GT). Subtumor parts are represented as follows: green, edema; blue, enhancing tumor; red, necrosis. BraTS, Brain Tumor Segmentation; FLAIR, fluid-attenuated inversion recovery.

Since 2012, every year, the BraTS challenge is organized at Medical Image Computing and Computer Assisted Intervention (MICCAI) conference (Menze et al., 2015). The challenge is to segment HGG and low-grade glioma (LGG) with high accuracy. From 2017 onwards, the task is extended for the prediction of OS of the patients in days as well (Bakas et al., 2017a,b, 2019). The BraTS organizers had provided multi-institutional training dataset of 163 patients diagnosed with GBM. For the validation dataset and test dataset, 53 and 130 cases were provided separately. The data were obtained from various institutions all over the globe, with different clinical protocols and scanners. For each patient, MRI data of size 240 × 240 × 155 were provided with FLAIR, T1, T1ce, and T2 modalities along with the ground truth as shown in Figure 1. The same annotation protocol was followed to segment all the cases manually by one to four raters, which were later verified by expert neurologists with more than 15 years of experience. The labels were termed as edema, enhancing tumor (ET), and necrosis. One of the tasks of BraTS 2018 challenge was to auto-segment the tumor into its three constituent regions, namely,

1. enhancing tumor region (ET), which shows hyperintensity in T1 postcontrast when compared with T1;
2. tumor core (TC), which entails the ET, necrotic (fluid filled), and non-enhancing (solid) parts; and
3. whole tumor (WT), which includes all intra-tumor parts along with edema.

**Figure 2:**
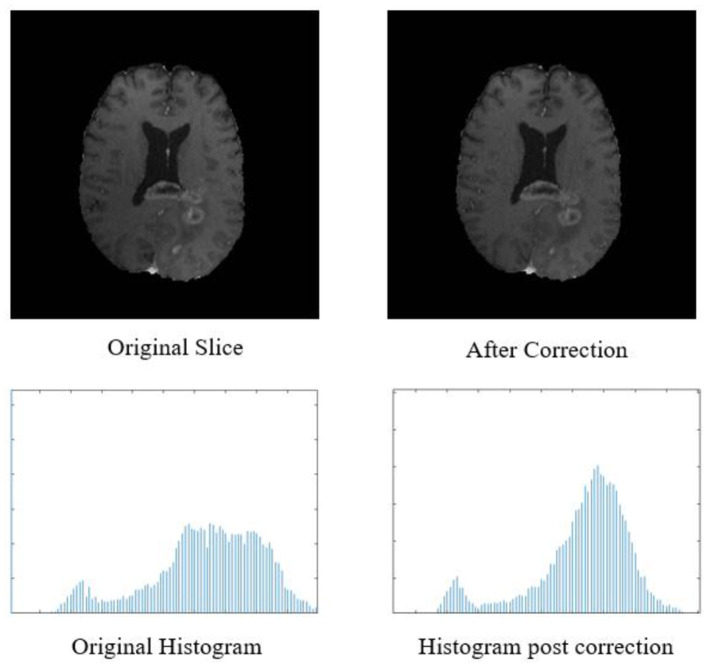
Top row: original input MR slice and slice after biased field correction. Bottom row: corresponding histograms of original slice and histogram after biased field correction. The horizontal *X*-axis of the histogram is intensity, and the vertical *Y* axis is frequency.

Additional information like resection status, age, and survival in days were also provided exclusively for OS prediction task. The MR data provided by BraTS organizers was skull stripped and co-registered to 1 mm × 1 mm × 1 mm isotropic resolution. The proposed three-step pipeline is shown in Figure 2.

### Proposed Methodology

**Figure 3:**
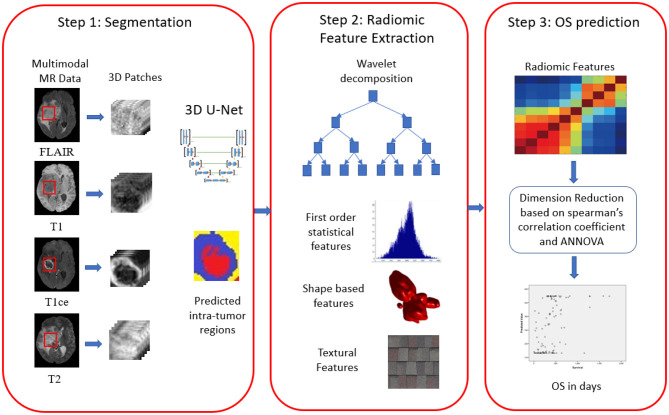
Proposed three-step framework for overall survival prediction in glioblastoma (GBM).

We proposed three-step methodology for OS prediction, as shown in Figure 3. In our approach, radiomic features were extracted on region of interest (ROI). The segmentation labels were provided for training dataset only, and hence, we segmented the tumors in validation and test dataset first and then extracted the radiomic features on the segmented ROI as first step. In step 2, radiomic features were extracted, and feature selection and OS prediction model was designed in step 3.

#### Preprocessing

The biased field algorithm was applied on FLAIR, T1, T2, and T1ce channel to correct the intensity inhomogeneity with N4ITK tool (Tustison et al., 2010). From Figure 2, it can be observed that the slice after bias field correction is more homogeneous in terms of intensity. All the four MR channels were normalized to zero mean and unit variance. We extracted multi-channel and multi-regional radiomic features with the help of intra-tumor annotations provided by the organizers on training dataset. We segmented the tumor with patch-based 3D U-Net architecture (Baid et al., 2019, 2020).

### Radiomic Feature Extraction

We extracted radiomic features on FLAIR and T1ce channels. Because whole tumor is best seen in FLAIR modality and enhancing tumor boundaries can be best visualized in T1ce modality, we selected these channels only for feature extraction. We computed radiomic features on these modalities with three varying combinations of intra-tumor parts as a whole tumor, that is, all intra-tumor parts, necrosis with enhancing tumor, and enhancing tumor only. So at the end, we had six combinations to extract radiomic features.

**Figure 4:**
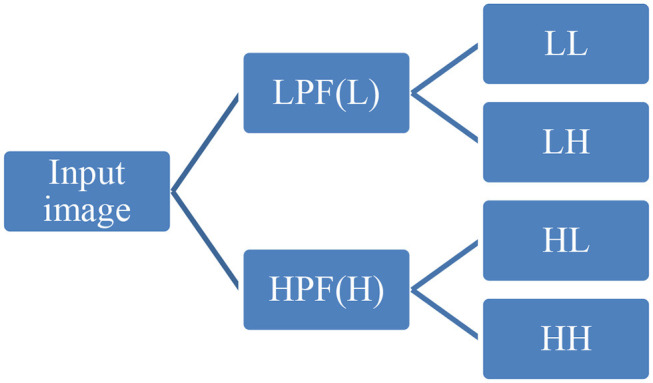
Representative diagram for stationary wavelet decomposition. LPF, low-pass filter; HPF, high-pass filter.

First-order statistical features and shape-based features were extracted from these combinations of ROI and MR channels. ROI was decomposed into four sub-bands with a multi-level 2-D stationary wavelet decomposition using a biorthogonal wavelet (Kickingereder et al., 2016b). In the first step, ROI was decomposed into two sub-bands with low-pass filter (LPF) and high-pass filter (HPF). Further, these sub-bands were again passed through LPF and HPF, giving LL, LH, HL, and HH bands (Figure 4). This was to extract directional texture features from approximate, horizontal, vertical, and diagonal components obtained after decomposition of the ROI (Nason and Silverman, 1995). It should be noted that we have not down-sampled the LPF and HPF to generate LL, LH, HL, and HH purposefully to avoid any sort of loss of information. GLCM features were extracted from these sub-bands (Haralick and Shanmugam, 1973). One hundred and thirteen first-order statistics, shape-based, and GLCM features were extracted for each tumor part and modality considering all four wavelet sub-bands. Thus, we had a total of 678 radiomic features extracted from six different combinations of tumor parts and modalities. Each patient in the BraTS dataset was provided with age as additional information, which we had concatenated in our feature vector. Finally, for each patient, we had 679 variables to be used to train the regression model for the survival prediction task. In training dataset, we had 163 patients for whom OS was provided in days. The radiomic feature extraction pipeline is available at Github^1^.

### Survival Prediction

Survival prediction was divided into two tasks. One task aimed at classifying patients into three survival groups obtained by unsupervised two-step clustering. These groups roughly correspond to the known survival groups in GBM (PMID: 22517216). The survival groups were characterized as long survivors (e.g., >900 days), short survivors (e.g., <300 days), and mid-survivors (e.g., between 300 and 900 days). For precise treatment planning, it is valuable to categorize a patient to either of these survival subgroups. This will enable clinicians to decide how aggressively a patient needs to be treated. The second task aimed at predicting OS in days, which is the same as the task required for BraTS 2018.

#### Delineating Survival Groups

**Figure 5:**
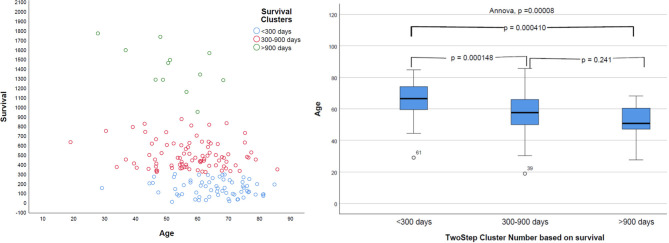
Distribution of survival groups identified using two-step clustering and correlation with age. Left: the age is in years and overall survival in given days. Right: on the *X*-axis of two-step cluster, Group 1, Group 2, and Group 3 are represented.

Natural grouping of patients based on survival was investigated using unsupervised two-step hierarchical clustering. This resulted in three groups with a good silhouette of separation: Group 1 (short survivors; patients with <300 days' OS, *n* = 65), Group 2 (mid-survivors; patients with OS between 300 and 900 days, *n* = 86), and Group 3 (long survivors; patients with >900 days' survival, *n* = 12). Pearson's correlation revealed a strong inverse relationship of age with this group, with younger patients having the greatest OS (*p* = 0.000008, *r* = −0.368), as shown in Figure 5.

#### Assessment of Relationship With Survival and Radiomic Feature Vector Dimension Reduction

In order to reduce the dimensionality of the feature vector, Spearman's correlation coefficient was calculated for each pair of radiomic features. The features having Spearman's correlation coefficient >0.95 with each other were discarded, retaining a single feature in each set (Supplementary Table 1). This reduced the feature vector size from 679 to 118. The feature set was further reduced to 54 by excluding all variables with statistically insignificant (*p* > 0.05) relationship with the survival groups (tested using ANOVA) identified above and with OS (tested using Pearson's correlation coefficient). It was observed that in terms of normalized importance, age is the most important feature. Because whole tumor is visible in FLAIR modality with hyperintense pixels, their features followed age. The enhancement tumor and core tumor counts were of significant importance for survival prediction (Supplementary Table 1).

#### Predicting Survival Groups Using Radiomic Features

Neural networks were designed using MLP to build a predictive model using the reduced radiomic feature vector set and age.

##### Neural Network Design

A single neural network was designed to classify the features in the three survival categories and to predict the OS in days. The neural network designed had two hidden layers. The number of units per layer were fixed to “auto.” The sigmoid activation function was used in hidden layers and output layers. Results were replicated by setting a random seed. For fair evaluation and to avoid overfitting, the BraTS training dataset was further divided into training (51.5%), validation (14.7%), and testing (33.7%) subsets by using randomly generated Bernoulli variates. All the features were rescaled with an adjusted normalized correction of 0.2. We also performed an individual variable importance analysis (Supplementary Table 1).

## Result And Discussion

### Neural Network Performance

**Figure 6:**
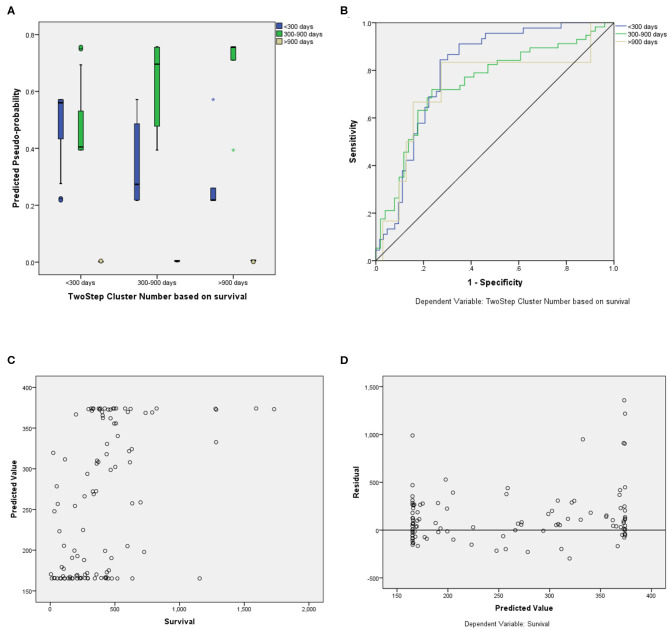
Performance summary of the overall survival (OS) prediction algorithm. **(A)** Predicted pseudoprobabilities across the three prediction categories. **(B)** Area under the receiver operating characteristic curve (AUC) for the three categories. **(C)** Dot plot of predicted and actual survival in days. **(D)** Residual vs. predicted plots for the survival prediction in days.

For the prediction of survival categories, the neural network demonstrated an accuracy of 70.2% in the training subset and 62.5 and 63.6% in the validation and testing subsets, respectively, which we divided from BraTS training dataset. The accuracy was 73% for the entire training dataset. The AUC was 0.799 (0.817 for Group 1, 0.709 for Group 2, and 0.784 for Group 3). A summary of the model performance is shown in Figure 6. The designed model performed better for patients in the mid-survivor groups, with the least accuracy for patients in the long-survivor group.

For fair evaluation of all the proposed algorithms of researchers participating in the BraTS challenge, organizers had provided an online evaluation platform. Participants were expected to submit the results on this platform, and later, they could download the quantitative results for the same^2^. It had been observed that despite less accuracy on validation dataset, our method achieved the third position in OS prediction task in the BraTS challenge^3^. The most convincing reason behind this was that all other participants might have overfitted their methods to the validation dataset. BraTS organizers have provided leaderboard of all the participants with segmentation and OS prediction task with several quantitative evaluation matrices^4^. We evaluated the proposed approach on BraTS training testing and validation dataset as shown in Table 1. The comparison between random forest and MLP is given in Table 2 on BraTS validation dataset.

**Table 1:** Quantitative evaluation of multilayer perceptron for OS prediction on the BraTS dataset.

**BraTS**	**Accuracy**	**MSE**	**Median**	**Std. deviation**	**Spearman *R***
**dataset**			**SE**		
Training	0.695	18,920.841	9,139.551	22,253.812	0.877
Validation	0.571	59,550,213.1	1,136,111.6	128,250,465.8	0.427
Testing	0.558	338219.366	38408.16	939986.796	0.222

*OS, overall survival; BraTS, Brain Tumor Segmentation; MSE, mean squared error*.

**Table 2:** Quantitative evaluation of MLP and RF for OS prediction on BraTS validation dataset.

**Approach**	**Accuracy**	**MSE**	**Median SE**	**Std. deviation**	**Spearman *R***
RF	0.375	6,109,105.6	47,545.13	143,070.37	0.11
MLP	0.571	5,955,021.1	11,361.6	12,825,046.8	0.427

*MLP, multilayer perceptron; RF, random forest; OS, overall survival; BraTS, Brain Tumor Segmentation; MSE, mean squared error*.

**Figure 7:**
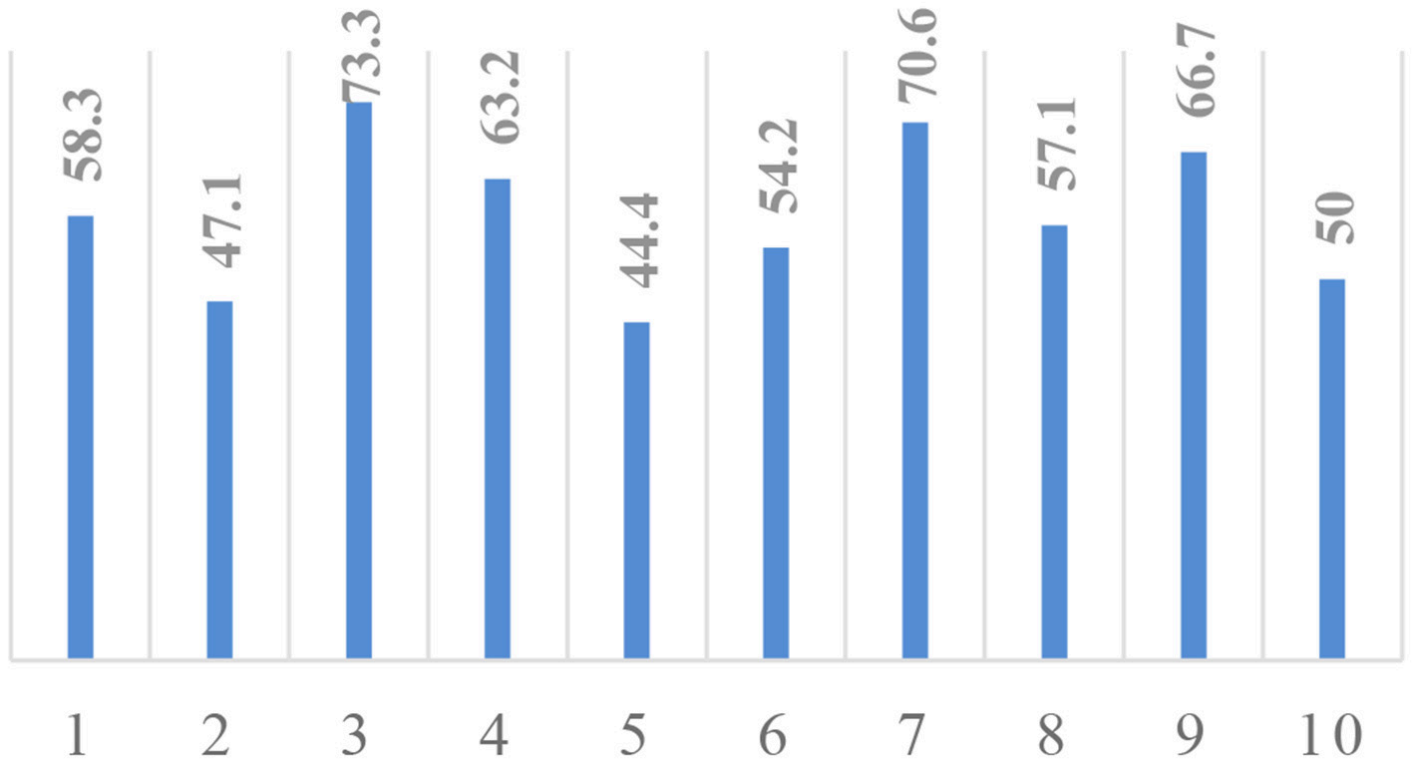
*K*-fold cross-validation analysis. *X*-axis, fold number; *Y*-axis, accuracy.

We have also evaluated the efficiency of the proposed approach with 10-fold validation. At every fold, 90% of patients are used for training and 10% of samples are kept as a holdout. The classification accuracy at each fold is given in Figure 7. The *X*-axis of the plot represents the fold number, and *Y*-axis gives the corresponding classification accuracy. The average accuracy is found to be 58.49, which is comparable with accuracy on BraTS 2018 validation dataset, which proves the robustness of the proposed approach.

## Discussion

Predicting outcomes has been the holy grail of modern oncology, notoriously difficult to achieve with high accuracy, yet driving numerous investigators toward finding newer ways of attempting to reach that goal. Because of multiple challenging factors, this task is out of clinical reach. Some of the challenges are the limitations of the human mind and recording devices to quantify the biological variations. The other is an amalgamation of cross-disciplinary interactions of clinical sides (for treatment planning) and engineering sides (toward quantitative analysis).

In this work, we have evaluated a couple of methods (MLP and RF) to predict OS using radiomic feature extracted using deep learning-based segmentation and feature pipeline as shown in Figure 3. What is interesting is the fact that although our segmentation pipeline did not feature in the top models submitted to BraTS 2018, our survival prediction pipeline did make it to the third position on the basis of the performance metrics decided by the BraTS 2018 organizers. The neural network achieved an accuracy of 0.583 with a relatively low standard error. Age was the most important variable in the predictive model. Further, we have also identified radiomic features that contributed maximally to the model.

The importance of the independent variable in descending order is given in Supplementary Table 1. It was observed that age is the most important factor for the OS prediction. We observed that radiomic analysis of tumor core region, which is comprised of necrosis and enhancing tumor on FLAIR modality contributed significantly toward prediction OS. It can be concluded that the core tumor count, that is, volume of enhancing tumor and tumor core, are of extreme importance in OS prediction of patients with GBM.

The amount of clinical information (only age and OS) provided in BraTS 2018 is extremely limited; and no details regarding gender, other co-existing comorbidities, performance status, and details of treatment received are provided. Considering these limiting factors, it is interesting to note that radiomic features coupled with age could explain a significant amount of variability seen in the OS of these patients with GBM. Although predicting survival in terms of closest number of days is desirable, in actual clinical practice, it often suffices to predict prognostically relevant groups for treatment intensification. For example, we were able to identify patients with <300 days of survival with a significantly high accuracy (0.804). These patients are ideal candidates for treatment intensification. Our accuracy in predicting survival for the long survivors was the least, possibly owing to the small number of cases in that group.

### Implementation Details

The radiomic feature extraction pipeline is designed in MATLAB environment. The neural network design, feature reduction, and other statistical analysis were performed with SPSS v24 on computing machine with Windows 10 operating system.

## Conclusion and Future Work

In this study, we evaluated the efficiency of radiomic features and machine learning-based classifier to predict the OS of the patients diagnosed with GBM. Multi-modal radiomic features were extracted from the FLAIR and T1ce channel of preoperated MRI data. OS of the patient was predicted with MLP and RF regressors. The classification accuracy shows that MLP outperformed over random forest in terms of accuracy. The proposed approach achieved the third position in BraTS 2018. We have also identified radiomic features that contribute maximally to the neural network's predictive ability. Further, the work could potentially include incorporating additional prognostic variables such as pathologic assessment information, molecular aberration information, comorbidities, and performance status into the predictive model.

## Data Availability Statement

Publicly available datasets were analyzed in this study. This data can be found here: The datasets analyzed for this study can be found in the BraTS 2018 dataset https://www.med.upenn.edu/sbia/brats2018/data.html.

## Author Contributions

UB, AM, and SR conducted the experiment. All authors contributed to writing the manuscript and are responsible.

## Conflict of Interest

The authors declare that the research was conducted in the absence of any commercial or financial relationships that could be construed as a potential conflict of interest.

## Footnotes

^1^https://github.com/ujjwalbaid0408/Radiomics

^2^https://ipp.cbica.upenn.edu/

^3^https://www.med.upenn.edu/sbia/brats2018/rankings.html

^4^https://www.cbica.upenn.edu/BraTS18/
