# Supplementary material for: Overall Survival Prediction in Glioblastoma With Radiomic Features Using Machine Learning
Source: Front Comput Neurosci. 2020 Aug 4;14:61. doi: 10.3389/fncom.2020.00061 (PMC7417437; doi:10.3389/fncom.2020.00061)
Supplement: Supplementary file 1 [file Data_Sheet_1.PDF]

Overall Survival prediction in Glioblastoma with Radiomic Features using Machine Learning

Supplementary Material

1 Importance of the independent variable in descending order

Feature	Modality	Region of Interest	Importance	Normalized Importance (%)
Age	--	--	0.07	100.0
Entropy	Flair	Nec+ET	0.049	70.3
Variance	Flair	Nec+ET	0.04	57.4
Enhance Count	Flair	Nec+ET	0.038	54.4
Core Count	T1ce	WT	0.034	49.10
Cluster Shade	T1ce	Nec+ET	0.031	44.7
Core Count	Flair	WT	0.030	43.4
Two step Cluster Number based on age	--	--	0.030	43.0~~%~~
Edema Count	Flair	Nec+ET	0.03	42.8
Dissimilarity	T1ce	Nec +ET	0.029	42.3
Core Count	Flair	ET	0.029	41.8
Difference in Entropy	Flair	ET	0.029	41.2

Enhance Count	Flair	WT	0.029	40.9
Variance	T1ce	Nec +ET	0.027	39.0
Maximum Probability	T1ce	ET	0.026	36.9
Sum of Variance	T1ce	ET	0.025	36.3
Homogeneity	T1ce	Nec +ET	0.024	34.7
Minimum	T1ce	WT	0.023	32.4
Correlation	T1ce	Nec +ET	0.022	31.0
Inverse Difference	T1ce	Nec +ET	0.021	30.0
Contrast	Flair	Nec +ET	0.021	29.9
Cluster Shade	T1ce	ET	0.019	26.7
Correlation	T1ce	Nec +ET	0.016	23.0
Variance	T1ce	ET	0.015	21.5
Maximum Probability	Flair	ET	0.013	19.0
Cluster Prominence	T1ce	ET	0.013	18.5
Dissimilarity	Flair	ET	0.013	18.5
Auto-Correlation	T1ce	Nec +ET	0.013	18.4
Inverse Difference	T1ce	ET	0.013	18.4
Sum of Squares Variance	T1ce	ET	0.012	17.3
Difference in Entropy	Flair	Nec +ET	0.012	17.0
Average	Flair	Nec +ET	0.012	16.6

Maximum Probability	T1ce	Nec +ET	0.011	15.4
Homogeneity	Flair	Nec +ET	0.01	14.7
Difference in Entropy	T1ce	Nec +ET	0.009	13.1
Mean	Flair	Nec +ET	0.009	12.4
Cluster Prominence	T1ce	Nec +ET	0.007	10.3
Sum Average	T1ce	Nec +ET	0.007	10.0
Inverse Difference	Flair	Nec +ET	0.006	9.2
Minimum	Flair	WT	0.005	7.8
Contrast	T1ce	Nec +ET	0.005	6.7
Sum of Intensities	Flair	ET	0.004	6.3
Contrast	Flair	ET	0.003	4.4
Homogeneity	Flair	ET	0.002	2.4
Contrast	T1ce	ET	0.001	1.9
Dissimilarity	T1ce	ET	0.001	1.7

2 Evaluate natural groupings (Two step clustering)

AGE

		Group 1 (<52 years)	Group 2 (52-65 years)	Group 3 (>65 years)
Count		37	67	59
Mean		43.53(1.23)	58.92(0.46)	72.47(0.65)
95% Confidence Interval for Mean	Lower Bound	41.03	58.01	71.18
	Upper Bound	46.03	59.83	73.77
5% Trimmed Mean		44.21	58.98	72.19
Median		46.26	59.58	71.37
Variance		56.28	13.93	24.68
Std. Deviation		7.5	3.73	4.97
Minimum		18.98	52.26	65.9
Maximum		51.76	64.86	85.76
Range		32.78	12.6	19.86
Interquartile Range		8.25	6.63	7.61
Skewness		-1.53(0.39)	-0.15(0.29)	0.69(0.31)
Kurtosis		2.29(0.76)	-1.29(0.58)	-0.16(0.61)

Survival

		Group 1 (<300 days)	Group 2 (301-900 days)	Group 3 (>900 days)
Count		65	86	12
Mean		147.45(10.31)	493.95(15.51)	1406.58(69.11)
95% Confidence Interval for Mean	Lower Bound	126.86	463.11	1254.48
	Upper Bound	168.03	524.8	1558.69
5% Trimmed Mean		146.86	484.79	1412.15

**Median**	147	458.5	1397.5
**Variance**	6902.94	20698.02	57309.72
**Std. Deviation**	83.08	143.87	239.39
**Minimum**	5	317	946
**Maximum**	296	871	1767
**Range**	291	554	821
**Interquartile Range**	127.5	226.75	305.25
**Skewness**	0.15(0.30)	0.90(0.26)	-0.22(0.64)
**Kurtosis**	-1.07(0.59)	-0.08(0.51)	-0.22(1.23)

3 Accuracy: For survival group classification

**Classification**					
Sample		Predicted			
		<300 days	300-900 days	>900 days	Percent Correct
Training	<300 days	25	8	0	75.8%
	300-900 days	12	34	0	73.9%
	>900 days	1	4	0	0.0%
	Overall Percent	45.2%	54.8%	0.0%	70.2%
Testing	<300 days	7	5	0	58.3%
	300-900 days	3	8	0	72.7%
	>900 days	0	1	0	0.0%
	Overall Percent	41.7%	58.3%	0.0%	62.5%
Holdout	<300 days	14	6	0	70.0%
	300-900 days	8	21	0	72.4%
	>900 days	1	5	0	0.0%
	Overall Percent	41.8%	58.2%	0.0%	63.6%
Dependent Variable: Two Step Cluster Number based on survival					
